# Comparison of different approaches to manage multi-site magnetic resonance spectroscopy clinical data analysis

**DOI:** 10.3389/fpsyg.2023.1130188

**Published:** 2023-04-20

**Authors:** Parker L. La, Tiffany K. Bell, William Craig, Quynh Doan, Miriam H. Beauchamp, Roger Zemek, Keith Owen Yeates, Ashley D. Harris

**Affiliations:** ^1^Department of Radiology, University of Calgary, Calgary, AB, Canada; ^2^Hotchkiss Brain Institute, University of Calgary, Calgary, AB, Canada; ^3^Alberta Children’s Hospital Research Institute, University of Calgary, Calgary, AB, Canada; ^4^Department of Pediatrics, Stollery Children’s Hospital, University of Alberta, Edmonton, AB, Canada; ^5^Department of Pediatrics, BC Children’s Hospital, University of British Columbia, Vancouver, BC, Canada; ^6^Department of Psychology, Ste-Justine Hospital Research Centre, University of Montreal, Montreal, QC, Canada; ^7^Department of Pediatrics and Emergency Medicine, Children’s Hospital of Eastern Ontario, University of Ottawa, Ottawa, ON, Canada; ^8^Department of Psychology, University of Calgary, Calgary, AB, Canada

**Keywords:** multi-site, multi-scanner, multi-vendor, statistical methods, concussion, ComBat harmonization, MR spectroscopy

## Abstract

**Introduction:**

The effects caused by differences in data acquisition can be substantial and may impact data interpretation in multi-site/scanner studies using magnetic resonance spectroscopy (MRS). Given the increasing use of multi-site studies, a better understanding of how to account for different scanners is needed. Using data from a concussion population, we compare ComBat harmonization with different statistical methods in controlling for site, vendor, and scanner as covariates to determine how to best control for multi-site data.

**Methods:**

The data for the current study included 545 MRS datasets to measure tNAA, tCr, tCho, Glx, and mI to study the pediatric concussion acquired across five sites, six scanners, and two different MRI vendors. For each metabolite, the site and vendor were accounted for in seven different models of general linear models (GLM) or mixed-effects models while testing for group differences between the concussion and orthopedic injury. Models 1 and 2 controlled for vendor and site. Models 3 and 4 controlled for scanner. Models 5 and 6 controlled for site applied to data harmonized by vendor using ComBat. Model 7 controlled for scanner applied to data harmonized by scanner using ComBat. All the models controlled for age and sex as covariates.

**Results:**

Models 1 and 2, controlling for site and vendor, showed no significant group effect in any metabolites, but the vendor and site were significant factors in the GLM. Model 3, which included a scanner, showed a significant group effect for tNAA and tCho, and the scanner was a significant factor. Model 4, controlling for the scanner, did not show a group effect in the mixed model. The data harmonized by the vendor using ComBat (Models 5 and 6) had no significant group effect in both the GLM and mixed models. Lastly, the data harmonized by the scanner using ComBat (Model 7) showed no significant group effect. The individual site data suggest there were no group differences.

**Conclusion:**

Using data from a large clinical concussion population, different analysis techniques to control for site, vendor, and scanner in MRS data yielded different results. The findings support the use of ComBat harmonization for clinical MRS data, as it removes the site and vendor effects.

## 1. Introduction

As with many imaging modalities, multiple sites and scanners are used to increase sample sizes and to best sample and represent the population in magnetic resonance spectroscopy (MRS) studies. However, scanner effects are substantial; both between and within vendor effects can affect MRS data (Near et al., 2013; Považan et al., 2020; Harris et al., 2022). Additionally, scanner updates and upgrades occur on different timelines at different sites, further increasing the variability in measures from each scanner/vendor/site. Given the increasing number of multi-site studies (Volkow et al., 2018, 2020), a better understanding of how best to account for scanner differences is needed, as these effects can subsequently influence results and their interpretation.

It is common to control for site-related variance within statistical models. Another method is to harmonize data, for example, using ComBat (Harris et al., 2022). ComBat is a harmonization approach originally developed for genetic data (Johnson et al., 2007), which has shown promise for removing site and scanner effects in MR imaging data, having been applied to structural imaging (Fortin et al., 2018), diffusion imaging (Fortin et al., 2017), and functional MRI (fMRI) (Yu et al., 2018) data. More recently, ComBat harmonization was applied to MRS data from 20 different sites in a healthy population (Bell et al., 2022). To our knowledge, no one has examined and compared approaches to account for multi-scanner MRS data in a clinical pediatric population.

To validate and compare approaches to account for multi-site data, one approach is to collect data on the same individuals across all scanners used in the main study (i.e., traveling subjects). While ideal, this experimental design needs to include many control participants scanned at each site (Maikusa et al., 2021) to validate approaches to account for multi-site effects. Given that the motivation for multi-site studies is generally to increase the sample size by recruiting at multiple cities, this becomes prohibitively expensive. Furthermore, the true amount of intra-individual variation is unknown. A similar challenge arises in comparing models to account for site; there is no known truth as to the level of site/scanner variance in the results, so comparing performance between models is challenging. Thus, the replication of findings in different studies examining and comparing approaches to harmonization using available multi-site data is the best alternative in validating techniques to control for multiple sites and scanners.

Concussion is a clinical condition that is becoming increasingly prevalent, with many studies of concussion using MRS to determine biochemical alterations (Joyce et al., 2022). Group differences found in the concussion literature are often subtle or inconsistent (Sarmento et al., 2009; Henry et al., 2010; Maugans et al., 2012; Vagnozzi et al., 2013; Chamard et al., 2014; Schranz et al., 2018). Many of these studies cite sample size as a limitation, and thus multi-site studies are a desirable response to increase recruitment, as with many other conditions.

Using data from a pediatric cohort including patients with concussion or orthopedic injury (OI) across five sites, we compared the use of GLM models and linear mixed-effects models, controlling for scanner, site, and vendor, to ComBat harmonization. ComBat was first validated on MRS data by Bell et al. (2022), who used a healthy control, adult dataset that included 20 scanners across 20 sites with a maximum of 12 datasets from each site. We expand on this in the current manuscript, by comparing approaches to account for site when examining differences between pediatric concussion and OI groups. In concussion research, OI is used as a comparison group to determine head injury-specific effects rather than general effects of injury (Yeates et al., 2017). Furthermore, the data here are from five cities with six scanners, and they therefore span few sites, each with more data, compared to the many sites, each contributing fewer datasets, in Bell et al. (2022). Differences in data distribution may influence the efficacy of accounting for site using either ComBat harmonization or different statistical approaches.

For clarity, here site refers to a research center in a single city, vendor is the scanner manufacturer, and scanner is an individual MRI machine (i.e., a single site may have multiple scanners).

## 2. Materials and methods

### 2.1. Participant recruitment

The data for the current study included 545 MRS datasets (361 concussion 184 OI) acquired across the five sites and six scanners (one site had two scanners). A total of 287 participants were scanned with GE scanners and 258 with Siemens scanners. The parent study was designed to better understand pediatric concussion [Advancing Concussion Assessment in Pediatrics (A-CAP)] and included two groups: participants with concussion and a comparison group with OI. Recruitment occurred at five emergency departments in Canada (Alberta Children’s Hospital, Calgary; Stollery Children’s Hospital, Edmonton; British Columbia Children’s Hospital, Vancouver; Children’s Hospital of Eastern Ontario, Ottawa; and Centre Hospitalier Universitaire Sainte-Justine, Montreal), with an overall goal of 700 concussion participants and 300 OI participants within 2 years. After the initial recruitment, the eligible participants returned 1–2 weeks following injury for an assessment that typically included an MRI scan with an MRS acquisition.

Briefly, all the participants were from 8 to <17 years of age. The concussion participants were defined as children who had sustained a blunt head trauma and presented with at least one of the following characteristics: an observed loss of consciousness, a Glasgow Coma Scale (GCS) score of 13 or 14, or at least one acute sign or symptom of concussion. Signs of more severe traumatic brain injury resulted in exclusion from the sample. The OI participants were defined as children who sustained either an upper or lower extremity fracture, sprain, or strain due to blunt force resulting in an Abbreviated Injury Scale (Association for the Advancement of Automotive Medicine, 2016) score of four or less. The OI participants were excluded if there was any head trauma, suspicion of concussion, or an injury requiring surgical intervention or procedural sedation at the time of recruitment. For detailed information on the parent study protocol, inclusion/exclusion criteria, and other data not used in this study, see Yeates et al. (2017). The study was approved by the research ethics board at each participating site, and informed consent and assent was obtained from the parents/guardians and the youth participants, respectively.

### 2.2. Magnetic resonance imaging and magnetic resonance spectroscopy

All imaging was performed at 3T. A T1-weighted anatomical acquisition was acquired for voxel placement and tissue segmentation. Sites using a Siemens scanner acquired a 3D T1-weighted magnetization-prepared rapid acquisition gradient echo (MPRAGE) with TR/TE/TI = 1,880/2.9/948 ms and a field of view of 25.6 cm^2^. Sites using a GE scanner acquired a 3D T1-weighted fast spoiled gradient echo brain volume (FSPGR BRAVO) image with a TR/TE/TI = 8,250/3.16/600 ms with a field of view of 24 cm^2^. Both acquisitions used 192 slices, a flip angle of 10°, and had a voxel size of 0.8 mm × 0.8 mm × 0.8 mm.

A short echo time point-resolved spectroscopy (PRESS) sequence was used at all sites. PRESS was chosen as it was available at all sites; it is also consistent with recent recommendations from the ENIGMA MRS working group in traumatic brain injury (Bartnik-Olson et al., 2021). The following parameters were used in the PRESS acquisition: TE/TR = 30 ms/2,000 ms, 96 water suppressed averages, eight unsuppressed water averages, spectral width of 5,000 Hz (GE) or 2,000 Hz (Siemens), and number of points = 4,096 (GE) or 2,048 (Siemens). This study placed a 2 cm × 2 cm × 2 cm voxel in the left dorsal lateral prefrontal cortex (L-DLPFC). Each site was provided with reference images for a standardized voxel placement. Example voxel placement is shown in Supplementary Figure 1. The minimum reporting standards for *in vivo* MRS studies is included in Supplementary Table 1.

### 2.3. MRS data analysis

As the GE data had individual averages available, the pre-processing pipeline included the following: combination of receiver channels, removal of bad averages, retrospective shot-by-shot frequency and phase correction, left shifting, and zero-order phase correction, following the consensus of best practices (Near et al., 2020). These pre-processing steps were automated and completed using FID-A (Simpson et al., 2017). The Siemens data only had the fully averaged scan; therefore, the only pre-processing performed was by the vendor software prior to export of the data.

The data were then quantified relative to water with LCModel version 6.3-1J (Provencher, 2001), which includes eddy-current correction and water scaling. Customized basis sets for each vendor were generated in FID-A using specific pulse shapes and relevant parameters (e.g., spectral width and number of points), and they included the following metabolites: alanine, aspartate, β -hydroxybutyrate, choline, citrate, creatine (Cr), ethanol, gamma-aminobutyric acid (GABA), glucose, glutamine, glutamate, glycine, glycerophosphocholine, glutathione, *myo*-inositol, lactate, *N*-acetyl-aspartate (NAA), *N*-acetyl-aspartyl-glutamate (NAAG), phosphocholine, phosphocreatine (PCr), phosphoenolamine, *scyllo*-inositol, and taurine. The default macromolecular and lipid basis sets were also included in the LCModel analysis.

Finally, outputs from LCModel were corrected for tissue-specific relaxation and water visibility according to recommendations and guidelines (Near et al., 2020). For each MRS voxel, coregistration to the individual’s corresponding anatomical T1-weighted image and segmentation into white matter (WM), gray matter (GM), and cerebrospinal fluid (CSF) were performed using the function “CoRegStandAlone” in Gannet (Version 3.1) (Edden et al., 2014). Metabolite quantification accounting for tissue-specific T1- and T2-relaxation and water density was determined using the equations specified in Gasparovic et al. (2006), with values for 3T taken from Gasparovic et al. (2018), as recommended by the expert consensus (Near et al., 2020). Example spectra from each scanner are included in Supplementary Figure 1.

### 2.4. Quality control

Spectral quality was first assessed with visual inspection by one analyzer (PLL), and a second analyzer (ADH) assisted in borderline decisions for quality. Quantitative measures of quality included the linewidth (the full-width half-maximum, FWHM) of the water peak and the signal-to-noise ratio (SNR) of the NAA peak, both determined from FID-A. Spectral fitting quality was assessed using Cramer-Rao lower bounds generated in LCModel. The thresholds for quality were an SNR of at least 45 and a Cramer-Rao lower bound of less than 20% for each metabolite.

### 2.5. Single-site effects

There is no gold standard available to evaluate the performance of each approach to account for site/scanner effects. However, the performance can be partially evaluated by the consistency between the results of the full dataset (after accounting for site/scanner) and the results of each site independently. The effect size (Cohen’s d) of the group (i.e., the effect size of concussion vs. OI) was calculated for four of the sites to serve as a reference for expected group differences in the entire sample when accounting for site. Additionally, the effects of age and sex were explored at the four sites to serve as a reference for their effects on metabolites at each site. The Montreal site was excluded from single-site analyses due to the small sample size, which was also split between the two scanners used at this site (GE/Siemens *n* = 29/19), and the disproportionate sample size between the two groups (concussion/OI *n* = 37/11).

### 2.6. Multi-site metabolite level comparisons

In testing for group differences, seven approaches to control for site and vendor effects were compared for the five metabolites of interest: tNAA, tCr, tCho, Glx, and mI (here, we describe non-harmonized data using ComBat as metabolite concentrations). Age and sex were controlled for in all analyses either as covariates in the general linear model (GLM) or as fixed effects (Linear Mixed Models). Between model fits were compared by using the Akaike information criterion (AIC) for the GLM’s and the mixed models.

Model 1: GLM model including covariates for vendor (GE or Siemens) and site (five sites) applied to the metabolite concentrations.
Metabolite concentration ∼ (Group) + (Age) + (Sex) + (Site) + (Vendor).
Model 2: Linear mixed-effects model including group as a fixed effect, while site and vendor are included as random effects on metabolite concentrations.
Quantified Metabolite concentration ∼ (Group) + (Age) + (Sex) + Random_(*Site*)_ + Random_(*Vendor*)_.
Model 3: GLM model including a covariate for scanner (six in total) applied to the metabolite concentrations.
Metabolite concentration ∼ (Group) + (Age) + (Sex) + (Scanner).
Model 4: Linear mixed-effects model including group as a fixed effect, while scanner is included as a random effect on metabolite concentrations.
Metabolite concentration ∼ (Group) + (Age) + (Sex) + Random_(*Scanner*)_.
Model 5: GLM model including a covariate for site applied to harmonized metabolite concentrations by vendor using ComBat.
Metabolite Concentrations Harmonized by Vendor ∼ (Group) + (Age) + (Sex) + (Site).
Model 6: Linear mixed-effects model including group as a fixed effect, while site is included as a random effect on harmonized metabolite concentrations by vendor using ComBat.
Harmonized Metabolite Concentrations by Vendor ∼ (Group) + (Age) + (Sex) + Random_(*Site*)_.
Model 7: GLM model applied to harmonized metabolite concentrations by scanner using ComBat.
Harmonized Metabolite Concentrations by Scanner ∼ (Group) + (Age) + (Sex).

ComBat harmonization was performed on MRS data using the neuroComBat function (version 1.0.5 available at https://github.com/Jfortin1/ComBatHarmonization/tree/master/R) in R (version 4.0.4), as performed by Bell et al. (2022). ComBat operates by estimating an empirical statistical distribution for each parameter to correct for a chosen covariate while maintaining the variance from other covariates. It does this by applying a linear mixed-effects regression with terms for variables of non-biological effect (Fortin et al., 2017). For MRS data, the individual quantified metabolite concentrations (tNAA, tCr, tCho, Glx, and mI) are harmonized separately according to the vendor/scanner.

Two follow-up analyses to test the effectiveness of ComBat harmonization to remove the vendor and scanner effects were completed for Models 5 and 7, with vendor and scanner included as covariates in the respective GLMs. A follow-up analysis to test the effectiveness of ComBat harmonization on removing scanner-related effects in the linear mixed-effects models was completed. All statistical analyses were completed using IBM SPSS 26 (IBM Corp Released, 2019; IBM SPSS Statistics for MacOS, Version 26.0. IBM Corp., Armonk, NY, USA).

## 3. Results

### 3.1. Demographics

The concussion group was composed of 62% male participants, and the average age was 12.31 ± 2.46 years. The OI group was 55% male and had an average age of 12.57 ± 2.19 years. The groups did not significantly differ in age and sex.

### 3.2. MRS data characteristics

The SNR (measured using the NAA peak) had a similar range across the injury groups, but it was significantly higher in the concussion group compared to the OI group (*p* = 0.009). The mean SNR of GE/Siemens data was 82.8/185, and the FWHM was 10.6/8.34 Hz. A full breakdown of the FWHM and SNR of each site is displayed in Supplementary Table 2. Additionally, the mean and standard deviations of all metabolites between the different sites is presented in Supplementary Table 3.

When examining individual sites, no significant group differences were found for any metabolite, as previously reported (La et al., 2023). Given that the group comparison (concussion vs. OI) was not significant at the four sites with the largest recruitment, and these group comparisons had small effect sizes, we assume there are no significant group differences in all metabolites (Table 1).

**TABLE 1:** Effect size estimates (Cohen’s d) of the comparison between concussion and orthopedic injury (OI) groups for each metabolite at the four largest sites (Calgary, Edmonton, Ottawa, Vancouver).

		tNAA	tCr	tCho	Glx	mI
**Site (vendor)**	***N***	**d**	**d**	**d**	**d**	**d**
Calgary (GE)	138	0.103	0.133	-0.105	-0.019	-0.247
Edmonton (Siemens)	125	-0.072	0.189	-0.069	-0.053	0.179
Ottawa (Siemens)	114	-0.417	-0.482	-0.125	0.167	-0.147
Vancouver (GE)	120	-0.182	-0.011	-0.172	-0.016	-0.065

Note that Montreal was excluded from these estimates due to different vendors within the same site.

When examining the age effects, we found that age was a significant covariate in tNAA analyses for two sites (*p* < 0.05), and one site had a trend level significance (*p* = 0.053). Age was also significant in the Glx analyses for one site (*p* = 0.013), with trend level significance in one other site (*p* = 0.058). Sex was not a significant factor in any metabolite at any of the four sites (*p* > 0.05).

### 3.3. Model results

Model 1: The univariate GLM applied to the metabolite concentrations and including covariates for site and vendor showed no significant effect of group (concussion vs. OI) in any metabolite. The vendor was a significant factor for each metabolite, and the site was significant for tNAA, tCho, and Glx. Age was significant for tNAA and Glx. Sex was not significant in any metabolite models. Additional model details are shown below in Table 2. These results were previously reported in La et al. (2023). Each metabolite models’ fit was measured *via* the AIC value, as demonstrated in Table 2.

**TABLE 2:** Summary of the independent univariate general linear model (GLM) models for each metabolite (tNAA, tCr, tCho, Glx, and mI) to investigate group differences (concussion vs. OI) in the metabolite concentrations (Model 1).

	tNAA		tCr		tCho		Glx		mI	
	**AIC = −210.71**		**AIC = −507.86**		**AIC = −1,595**		**AIC = 721.4**		**AIC = −308.5**	
	**Estimate (SE)**	**t, *p***	**Estimate (SE)**	**t, *p***	**Estimate (SE)**	**t, *p* =**	**Estimate (SE)**	**t, *p* =**	**Estimate (SE)**	**t, *p* =**
**Covariates**										
Group	−0.007 (0.075)	−0.09, 0.928	0.027 (0.057)	0.48, 0.635	−0.026 (0.021)	−1.22, 0.223	0.096 (0.176)	0.545, 0.586	0.012 (0.069)	0.173, 0.863
Age	0.069 (0.015)	**4.6, 0.0001**	0.006 (0.011)	0.497, 0.619	−0.004 (0.004)	−0.95, 0.345	−0.117 (0.035)	**−3.34, 0.001**	0.0001 (0.014)	−0.03, 0.978
Sex	−0.009 (0.072)	−0.13, 0.896	−0.059 (0.055)	−1.08, 0.281	−0.003 (0.02)	−0.15, 0.882	0.325 (0.169)	1.93, 0.054	−0.008 (0.066)	−0.123, 0.902
Site	−0.065 (0.023)	**−2.8, 0.005**	0.03 (0.018)	1.73, 0.085	−0.025 (0.007)	**−3.77, 0.0001**	−0.128 (0.055)	**−2.35, 0.019**	0.016 (0.021)	0.753, 0.452
Vendor	−2.826 (0.071)	**−39.9, 0.0001**	−0.673 (0.054)	**−12.5, 0.0001**	−0.383 (0.02)	**−19.3, 0.0001**	−3.927 (0.167)	**−23.6, 0.0001**	−1.446 (0.065)	**−22.3, 0.0001**

The general linear model (GLM) includes covariates for age, sex, site (five sites), and vendor (two vendors). The *p*-values in bold were considered statistically significant at *p* < 0.05.

Model 2: The linear mixed-effects model showed that group was not significant for any metabolite and that age had significant effects on tNAA levels, while age and sex had significant effects for Glx levels (*p* < 0.05). The random effects of the site and vendor did not significantly impact the models (*p* > 0.05). The full model results are shown in Table 3. Each metabolite models’ fit was measured via the AIC value, as demonstrated in Table 3.

**TABLE 3:** Details of the linear mixed-effects model with metabolites of interest (tNAA, tCr, tCho, Glx, and mI) as the dependent variable.

	tNAA		tCr		tCho		Glx		mI	
	**AIC = 1,345**		**AIC = 1,043**		**AIC = −24.47**		**AIC = 2,233**		**AIC = 1,250**	
	**Estimate (SE)**	**t, *p***	**Estimate (SE)**	**t, *p***	**Estimate (SE)**	**t, *p***	**Estimate (SE)**	**t, *p***	**Estimate (SE)**	**t, *p***
**Fixed effects**										
Group	−0.02 (0.08)	−0.282, 0.778	0.01 (0.06)	0.239, 0.812	−0.03 (0.02)	−1.49, 0.138	0.05 (0.17)	0.274, 0.784	0.004 (0.07)	0.05, 0.957
Age	0.07 (0.01)	**4.87, 0.0001**	0.01 (0.01)	0.642, 0.521	−0.002 (0.004)	−0.685, 0.493	−0.11 (0.03)	**−3.24, 0.001**	−0.0003 (0.01)	−0.03, 0.980
Sex	0.003 (0.07)	0.036, 0.97	0.05 (0.05)	0.966, 0.335	−0.00009 (0.02)	−0.01, 0.996	−0.35 (0.16)	**−2.19, 0.029**	0.006 (0.07)	0.09, 0.930
	**Estimate (SE)**	***P***	**Estimate (SE)**	***P***	**Estimate (SE)**	***P***	**Estimate (SE)**	***P***	**Estimate (SE)**	***P***
**Random effects**										
Site	0.03 (0.03)	0.280	0.02 (0.02)	0.254	0.003 (0.002)	0.262	0.62 (0.48)	0.197	0.003 (0.006)	0.649
Vendor	4.08 (5.79)	0.481	0.22 (0.32)	0.494	0.06 (0.09)	0.486	8.11 (11.6)	0.485	1.03 (1.46)	0.481

The fixed effects were group (concussion vs. OI), age, and sex. Site and vendor were included as random effects in this model (Model 2). The *p*-values in bold were considered statistically significant at *p* < 0.05.

Model 3: The univariate GLM applied to the metabolite concentrations and including the scanner as a covariate showed significant group differences in tNAA and tCho levels. tCr and Glx did not demonstrate significant group differences, and while not significant, the group difference in mI approached significance (Table 4). The scanner was a significant factor for tNAA, tCho, and Glx. Age was significantly associated with tNAA and Glx, and sex had significant effects for Glx. The model details are shown in Table 4. Each metabolite models’ fit was measured via the AIC value, as demonstrated in Table 4.

**TABLE 4:** Summary of the independent univariate general linear model (GLM) models for each metabolite to investigate group differences (concussion vs. OI) in tNAA, tCr, tCho, Glx, and mI (Model 3).

	tNAA		tCr		tCho		Glx		mI	
	**AIC = 534.7**		**AIC = −372**		**AIC = −1,312**		**AIC = 1,105**		**AIC = 45.96**	
	**Estimate (SE)**	**t, *p* =**	**Estimate (SE)**	**t, *p* =**	**Estimate (SE)**	**t, *p* =**	**Estimate (SE)**	**t, *p* =**	**Estimate (SE)**	**t, *p* =**
**Covariates**										
Group	−0.372 (0.15)	**−2.52, 0.012**	−0.06 (0.064)	−0.93, 0.635	−0.075 (0.027)	**−2.75, 0.006**	−0.408 (0.25)	−1.64, 0.102	−0.176 (0.1)	−1.86, 0.063
Age	0.078 (0.03)	**2.64, 0.008**	0.008 (0.013)	0.63, 0.528	−0.003 (0.005)	−0.49, 0.624	−0.103 (0.05)	−**2.08, 0.038**	0.004 (0.019)	0.23,0.818
Sex	0.18 (0.14)	1.27, 0.206	−0.013 (0.062)	−0.20, 0.840	0.022 (0.026)	0.855, 0.393	0.588 (0.24)	**2.46, 0.014**	0.09 (0.09)	0.993, 0.321
Scanner	−0.08 (0.035)	**−2.26, 0.024**	0.019 (0.015)	1.25, 0.212	−0.022 (0.006)	**−3.38, 0.001**	−0.127 (0.06)	**−2.13, 0.033**	−0.002 (0.023)	−0.101, 0.920

The model includes covariates for age, sex, and scanner (six scanners). The *p*-values in bold were considered statistically significant at *p* < 0.05.

Model 4: The linear mixed-effects model showed that the group was not significant for any metabolite, and tNAA had significant age effects, while Glx had significant age and sex effects (*p* < 0.05). The random effect of the scanner did not significantly impact any metabolites (*p* > 0.05). The full model results are shown in Table 5. Each metabolite models’ fit was measured via the AIC value, as demonstrated in Table 5.

**TABLE 5:** Details of the linear mixed-effects model with metabolites of interest (tNAA, tCr, tCho, Glx, and mI) as the dependent variable.

	tNAA		tCr		tCho		Glx		mI	
	**AIC = 1,365**		**AIC = 1,051**		**AIC = −14.78**		**AIC = 2,246**		**AIC = 1,269**	
	**Estimate (SE)**	**t, *p***	**Estimate (SE)**	**t, *p***	**Estimate (SE)**	**t, *p***	**Estimate (SE)**	**t, *p***	**Estimate (SE)**	**t, *p***
**Fixed effects**										
Group	−0.03 (0.08)	0.132, 0.717	−0.36 (0.06)	0.21, 0.833	−0.03 (0.02)	−1.53, 0.127	0.04 (0.17)	0.242, 0.809	−0.003 (0.07)	−0.04, 0.968
Age	0.07 (0.01)	**23.5, 0.0001**	4.84 (0.01)	0.67, 0.501	−0.003 (0.004)	0.46, 0.463	−0.11 (0.03)	**−3.23, 0.001**	0.001 (0.01)	0.105, 0.917
Sex	0.003 (0.07)	0.002, 0.966	0.04 (0.05)	0.93, 0.353	−0.001 (0.02)	−0.07, 0.944	−0.35 (0.16)	**−2.19, 0.03**	−0.001 (0.07)	−0.02, 0.985
	**Estimate (SE)**	***P***	**Estimate (SE)**	***P***	**Estimate (SE)**	***P***	**Estimate (SE)**	***P***	**Estimate (SE)**	***P***
**Random effects**										
Scanner	2.47 (1.57)	0.116	0.15 (0.1)	0.133	0.04 (0.03)	0.121	5.4 (3.5)	0.119	0.6 (0.38)	0.12

The fixed effects were group (concussion vs. OI), age, and sex. Scanner was included as a random effect in this model (Model 4). The *p*-values in bold were considered statistically significant at *p* < 0.05.

**FIGURE 1:**
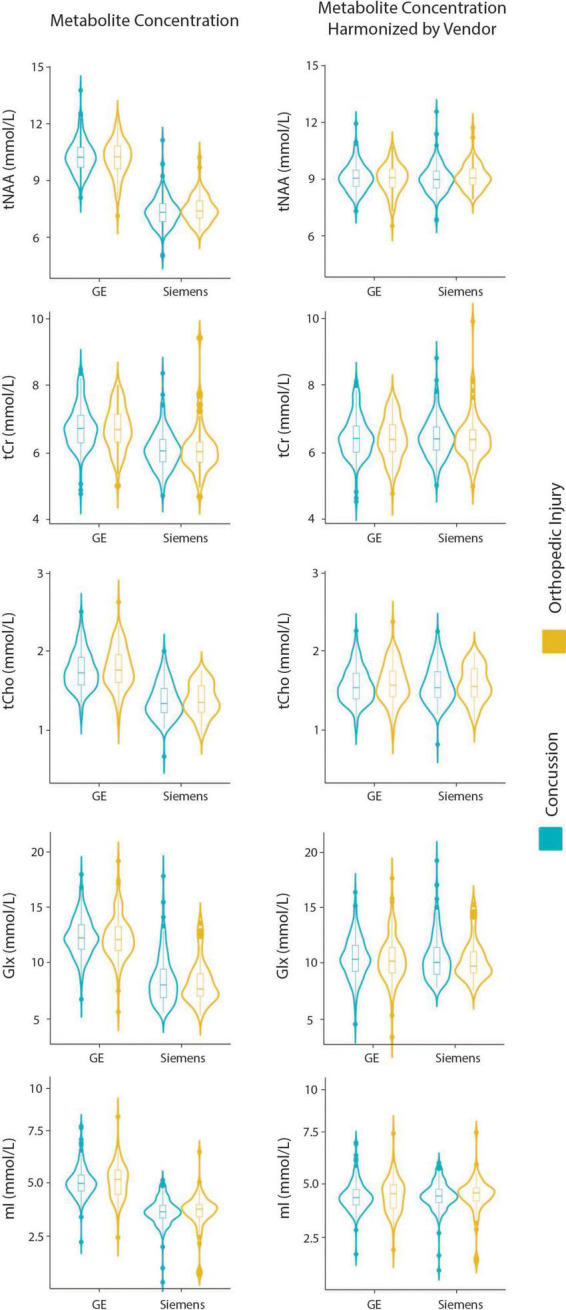
Violin plots showing metabolite concentrations pre- and post-ComBat harmonization by vendor (GE and Siemens) for tNAA, tCr, tCho, Glx, and mI. The data are separated by the two clinical groups: concussion and orthopedic injury.

Model 5: The univariate GLM applied to metabolite concentrations harmonized by the vendor showed no significant group differences in any metabolite. Site was a significant factor for tNAA, tCho, and Glx. Age was significant for the tNAA and Glx models. Sex did not significantly impact any metabolites in this model. Further model details are shown in Table 6. Each metabolite models’ fit was measured via the AIC value, as demonstrated in Table 6. Data pre- and post-harmonization by the vendor are presented in Figure 1. The follow-up GLM testing for vendor effects in the data harmonized by the vendor showed no significant effect of the vendor in any metabolite model, though the site was significant for tNAA and Glx (Table 7, Figure 1, and Supplementary Figure 2).

**TABLE 6:** Summary of the independent univariate general linear model (GLM) models for each metabolite to investigate group differences (concussion vs. OI) in tNAA, tCr, tCho, Glx, and mI.

	tNAA		tCr		tCho		Glx		mI	
	**AIC = −213.3**		**AIC = −512**		**AIC = −1,597**		**AIC = 715.4**		**AIC = −328.8**	
	**Estimate (SE)**	**t, *p* =**	**Estimate (SE)**	**t, *p* =**	**Estimate (SE)**	**t, *p* =**	**Estimate (SE)**	**t, *p* =**	**Estimate (SE)**	**t, *p* =**
**Covariates**										
Group	−0.01 (0.07)	−0.15, 0.881	0.03 (0.06)	0.442, 0.659	−0.02 (0.02)	−1.16, 0.248	0.091 (0.17)	0.523, 0.602	−0.01 (0.07)	−0.07, 0.942
Age	0.07 (0.02)	**4.66, 0.0001**	0.01 (0.01)	0.474, 0.635	−0.004 (0.004)	−0.92, 0.357	−0.1 (0.04)	**−3.41, 0.001**	0.0001 (0.01)	0.01, 0.993
Sex	−0.01 (0.07)	−0.097, 0.923	−0.06 (0.05)	−1.07, 0.284	−0.003 (0.02)	−0.17, 0.867	0.33 (0.17)	1.96, 0.051	−0.02 (0.06)	−0.27, 0.788
Site	−0.06 (0.02)	**−2.55, 0.011**	0.03 (0.02)	1.79, 0.074	−0.02 (0.01)	**−3.48, 0.001**	−0.15 (0.05)	**−2.7, 0.007**	0.01 (0.02)	0.69, 0.49

The data were harmonized by vendor using ComBat (two vendors) (Model 5). This model has covariates for age, sex, and site. The *p*-values in bold were considered statistically significant at *p* < 0.05.

**TABLE 7:** Summary of the independent univariate general linear model (GLM) models for each metabolite (tNAA, tCr, tCho, Glx, and mI) harmonized by vendor to investigate group differences (concussion vs. OI).

	tNAA		tCr		tCho		Glx		mI	
	**Estimate (SE)**	**t, *p* =**	**Estimate (SE)**	**t, *p* =**	**Estimate (SE)**	**t, *p* =**	**Estimate (SE)**	**t, *p* =**	**Estimate (SE)**	**t, *p* =**
**Covariates**										
Group	−0.01 (0.08)	−0.17, 0.862	0.03 (0.06)	0.457, 0.648	−0.03 (0.02)	−1.17, 0.242	0.1 (0.18)	0.51, 0.608	−0.01 (0.07)	−0.07, 0.946
Age	0.07 (0.02)	**4.66, 0.0001**	0.01 (0.01)	0.471, 0.638	−0.004 (0.004)	−0.92, 0.359	−0.12 (0.04)	**−3.41, 0.001**	0.0001 (0.01)	0.008, 0.994
Sex	−0.01 (0.07)	−0.08, 0.934	−0.06 (0.05)	−1.08, 0.281	−0.003 (0.02)	−0.15, 0.878	0.33 (0.17)	1.95, 0.051	−0.02 (0.07)	−0.27, 0.788
Site	−0.06 (0.02)	**−2.55, 0.011**	0.03 (0.02)	1.79, 0.074	−0.02 (0.01)	**−3.48, 0.001**	−0.15 (0.05)	**−2.69, 0.007**	0.01 (0.02)	0.69, 0.490
Vendor	0.01 (0.07)	0.203, 0.839	−0.01 (0.05)	−0.154, 0.878	0.004 (0.02)	0.2, 0.842	0.01 (0.17)	0.04, 0.967	−0.002 (0.06)	−0.03, 0.979

The model includes covariates for age, sex, site (five sites), and vendor (two vendors). This model shows that the effect of vendor is entirely removed with the inclusion of ComBat-harmonized data by vendor. The *p*-values in bold were considered statistically significant at *p* < 0.05.

Model 6: The linear mixed-effects model showed that the group was not significant for any metabolite, and tNAA had significant age effects, while Glx had significant age and sex effects (*p* < 0.05). The random effect of the site did not significantly impact any harmonized metabolite data (*p* > 0.05). The full model results are shown in Table 8. Each metabolite models’ fit was measured via the AIC value, as demonstrated in Table 8.

**TABLE 8:** Details of the linear mixed-effects model with metabolites of interest (tNAA, tCr, tCho, Glx, and mI) harmonized by vendor using ComBat as the dependent variable.

	tNAA		tCr		tCho		Glx		mI	
	**AIC = 1,342**		**AIC = 1,037**		**AIC = −28.69**		**AIC = 2,230**		**AIC = 1,228**	
	**Estimate (SE)**	**t, *p***	**Estimate (SE)**	**t, *p***	**Estimate (SE)**	**t, *p***	**Estimate (SE)**	**t, *p***	**Estimate (SE)**	**t, *p***
**Fixed effects**										
Group	−0.03 (0.07)	−0.35, 0.725	0.011 (0.06)	0.2, 0.841	−0.03 (0.02)	−1.4, 0.164	0.05 (0.17)	0.27, 0.788	−0.008 (0.07)	−0.12, 0.901
Age	0.07 (0.01)	**4.89, 0.0001**	0.007 (0.01)	0.63, 0.53	−0.003 (0.004)	−0.64, 0.521	−0.11 (0.03)	**−3.3, 0.001**	−0.0008 (0.01)	−0.06, 0.954
Sex	−0.0009 (0.07)	−0.01, 0.99	0.05 (0.05)	0.97, 0.331	0.001 (0.02)	0.05, 0.957	−0.36 (0.16)	**−2.2, 0.027**	0.02 (0.06)	0.27, 0.787
	**Estimate (SE)**	***P***	**Estimate (SE)**	***P***	**Estimate (SE)**	***P***	**Estimate (SE)**	***P***	**Estimate (SE)**	***P***
**Random effects**										
Site	0.02 (0.02)	0.281	0.02 (0.02)	0.247	0.002 (0.002)	0.252	0.57 (0.43)	0.192	0.0004 (0.004)	0.915

The fixed effects were group (concussion vs. OI), age, and sex. Site was included as a random effect in this model (Model 6). The *p*-values in bold were considered statistically significant at *p* < 0.05.

**FIGURE 2:**
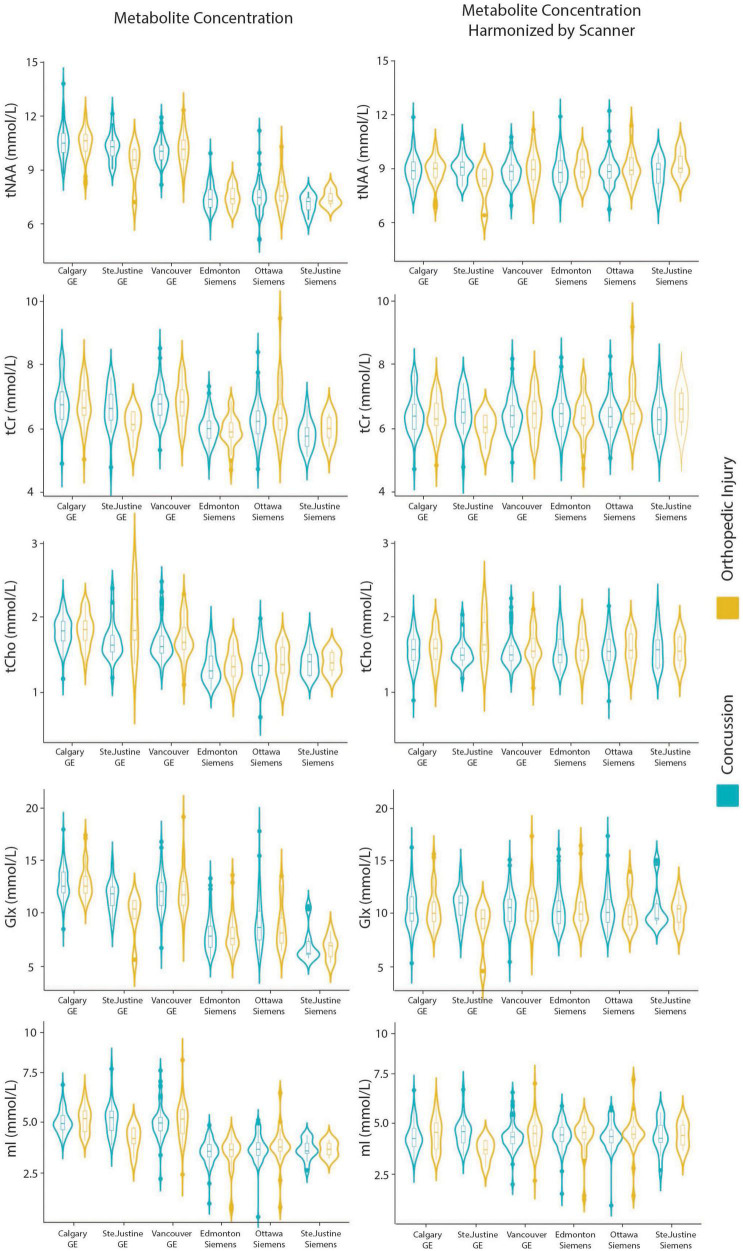
Violin plots showin gmetabolite concentrations and data harmonized by the six different scanners for tNAA, tCr, tCho, Glx, and mI. The data are separated by the two clinical outcome groups: concussion and orthopedic injury.

Model 7: The data harmonized by the scanner are shown in Figure 2. The univariate GLM applied to the data harmonized by the scanner showed no significant group differences for any metabolites. Age was significantly associated with tNAA and Glx levels. Sex was significantly related to Glx levels. Further model details are shown in Table 9. Each metabolite models’ fit was measured via the AIC value, as demonstrated in Table 9. The data both pre- and post-harmonization by the scanner are shown in Figure 2 and Supplementary Figure 2. In the follow-up analysis, data harmonized by the scanner had no scanner effects, as shown in Table 10, Figure 2.

**TABLE 9:** Summary of the independent univariate general linear model (GLM) models for each metabolite to investigate group differences (concussion vs. OI) in tNAA, tCr, tCho, Glx, and mI.

	tNAA		tCr		tCho		Glx		mI	
	**AIC = −235.6**		**AIC = −538.3**		**AIC = −1,614**		**AIC = 711.7**		**AIC = −338.1**	
	**Estimate (SE)**	**t, *p* =**	**Estimate (SE)**	**t, *p* =**	**Estimate (SE)**	**t, *p* =**	**Estimate (SE)**	**t, *p* =**	**Estimate (SE)**	**t, *p* =**
**Covariates**										
Group	−0.03 (0.07)	−0.43, 0.669	0.02 (0.1)	0.34, 0.737	−0.03 (0.02)	−1.36, 0.174	0.03 (0.17)	0.18, 0.855	−0.03 (0.07)	−0.398, 0.691
Age	0.08 (0.01)	**5.2, 0.0001**	0.01 (0.01)	0.65, 0.514	−0.002 (0.004)	−0.53, 0.594	−0.11 (0.03)	**−3.3, 0.001**	0.002 (0.01)	0.126, 0.900
Sex	−0.01 (0.1)	−0.09, 0.926	−0.06 (0.05)	−1.1, 0.267	0.003 (0.02)	0.132, 0.895	0.34 (0.16)	**2.1, 0.036**	−0.01 (0.06)	−0.1, 0.920

Metabolites were harmonized by scanner (six scanners) using ComBat (Model 7). This model has covariates for age and sex. The *p*-values in bold were considered statistically significant at *p* < 0.05.

**TABLE 10:** Summary of the independent univariate general linear model (GLM) models for each metabolite (tNAA, tCr, tCho, Glx, and mI) harmonized by scanner to investigate group differences (concussion vs. OI).

	tNAA		tCr		tCho		Glx		mI	
	**Estimate (SE)**	**t, *p* =**	**Estimate (SE)**	**t, *p* =**	**Estimate (SE)**	**t, *p* =**	**Estimate (SE)**	**t, *p* =**	**Estimate (SE)**	**t, *p* =**
**Covariates**										
Group	−0.03 (0.07)	−0.396, 0.692	0.02 (0.06)	0.34, 0.735	−0.03 (0.02)	−1.36, 0.173	0.03 (0.17)	0.165, 0.869	−0.03 (0.07)	−0.397, 0.691
Age	0.08 (0.02)	**5.2, 0.0001**	0.01 (0.01)	0.656, 0.512	−0.002 (0.004)	−0.54, 0.586	−0.11 (0.03)	**−3.3, 0.001**	0.002 (0.01)	0.12, 0.901
Sex	−0.01 (0.07)	−0.1, 0.936	−0.06 (0.05)	−1.1, 0.268	0.003 (0.02)	0.129, 0.897	0.33 (0.16)	**2.1, 0.037**	−0.01 (0.1)	−0.1, 0.920
Scanner	0.011 (0.02)	0.61, 0.545	0.001 (0.01)	0.07, 0.943	−0.001 (0.01)	−0.125, 0.901	−0.01 (0.04)	−0.34, 0.731	0.0001 (0.02)	−0.01, 0.995

The model includes covariates for age, sex, and scanner (six scanners). This model shows that the effect of scanner is entirely removed with the inclusion of ComBat-harmonized data by scanner. The *p*-values in bold were considered statistically significant at *p* < 0.05.

The follow-up analysis of a linear mixed-effects model of ComBat-harmonized data by the scanner, including scanner as a covariate, showed no significant effect of the scanner (*p* < 0.05), and the group also did not have a significant effect.

## 4. Discussion

In a large pediatric concussion and OI control dataset, we have demonstrated that different approaches to accounting for sites/scanners/vendors can affect MRS results and interpretation. Specifically, the GLM model testing for metabolite differences between groups (concussion vs. OI) that included scanner as a covariate (Model 3) showed a significant group effect for tNAA and tCho. tNAA and tCho were significantly lower in the concussion group compared to the OI group. Given the absence of a group effect in all other models and the analyses at each individual site, we conclude that group does not have a significant effect in this dataset and that the significant group effect seen when including scanner as a covariate (Model 3) was spurious. Additionally, the GLM Model 3 had the highest AIC for each metabolite in comparison to GLM models 1, 5, and 7, indicating a worse model fit. The linear mixed-effects models all performed similarly, although models 2 and 6 had similar AIC values, and model 4 had the largest AIC value. The best model determined from AIC appears to be GLM model 7 or the mixed-effects model 6, though similar results were also achieved in other models (Model 1 and Model 2). This work demonstrates that caution is needed when controlling for scanner/site/vendor, as this can have substantial implications on the results.

Despite the increasing standardization of imaging and MRS protocols, acquisition schemes can differ by vendor, for example, the pulse shapes and minimum achievable echo time (Harris et al., 2017, 2022). Furthermore, differences exist between scanners of the same vendor, for example, eddy currents and their impact on MRS data (Harris et al., 2022). These differences introduce the need to control for site, vendor, and/or scanner. For multi-site (multi-scanner) studies, controlling for site, vendor, and/or scanner as covariates is a common approach. While statistical theory suggests these methods should be effective to account for the variance associated with multiple scanners, our results suggest they can lead to erroneous results and interpretations. In this study, we found that controlling for scanner (i.e., the individual machine) within an GLM model produced different results than when controlling for site and vendor.

ComBat is a technique that harmonizes data for a chosen parameter (e.g., scanner) by estimating an empirical statistical distribution of multiple defined parameters (e.g., scanner, age, sex). It has the advantages of maintaining measures with meaningful values (quantified metabolite levels) and maintaining biological variability. Previous work in a healthy adult population of MRS data from 20 different sites and three different MRI vendors found that site and vendor effects were removed following ComBat harmonization (Bell et al., 2022). The current study also supports the use of ComBat for MRS data and extends these findings in a clinical pediatric population involving two different injury groups (concussion and OI). When harmonizing by vendor, a significant effect of site remained, though the effect of vendor was removed. This is perhaps not surprising given the known differences between MRS data collected on different scanners (Harris et al., 2022), and it supports the use of ComBat harmonization at the scanner level, unless the site effect is meaningful for a particular study. Replicating the results of Bell et al. (2022) in a different pediatric clinical dataset is important, as it confirms the utility of ComBat harmonization for MRS data, which is in line with recent commentaries on the importance of reproducibility in science (Stoddart, 2016; Kozlov, 2022).

In addition to removing site/scanner effects, it is important to maintain biological effects when harmonizing data. For that purpose, age and sex effects were examined in all the analyses. Some metabolites are known to be affected by development and are thus related to age (Cichocka and Bereś, 2018). tNAA increases with age in children and youth (Blüml and Panigrahy, 2013; Holmes et al., 2017; La et al., 2023), while Glx decreases with age in children and youth (Blüml and Panigrahy, 2013; Holmes et al., 2017; La et al., 2023). Overall, these expected age effects were seen in the individual site data. These age effects on tNAA and Glx were preserved in all seven models, including the ComBat-harmonized data. Other metabolites that were not related to age (tCr, tCho, and mI) retained non-significance in all the models. Glx was higher in male participants in Models 2, 3, 4, 6, and 7. Previous studies have reported sex effects in Glx (O’Gorman et al., 2011; Hädel et al., 2013), but these differences are not yet fully understood, and further studies are needed to confirm this relationship in pediatrics. In the single-site effect analyses, there were no sex-related effects observed.

### 4.1. Limitations and future directions

The current work has limitations. The first is that ComBat only allows for the harmonization of one factor at a time. In some cases, it is desirable to harmonize by more than one factor. For example, in our data, the site and vendor are two factors that were considered. To simultaneously address both, we used a combination variable, “scanner,” and controlled for it in the statistical analyses (GLM and mixed-effect models) and harmonized for it with ComBat. Secondly, ComBat uses the full dataset in the harmonization process, and new data cannot be added without performing harmonization on the new full dataset. This is because ComBat takes the empirical distribution of the full dataset and applies this to each sample. It is therefore not possible to add single datasets or to directly compare the numerical results of ComBat-harmonized data with other studies or datasets. Beyond these general limitations of ComBat, in this study, there were no group differences. While these results broadly suggest that caution is warranted in accounting for site/scanner effects, we cannot definitively conclude from this data that when true group differences exist, different approaches to account for multi-site/scanner effects could mask these effects. Regardless, the importance of thoroughly investigating the approach to account for multi-site/scanner effects remains an important finding, as erroneous interpretations may result. One recommendation for future studies is to investigate the consistency of the results when different approaches to account for site are used and also the consistency with the individual site data. In the future, machine learning may provide an alternative approach to harmonize or control for multi-site/scanner effects in MRS studies (Harris et al., 2022). Lastly, this work is limited to a single clinical research study; it contrasts two groups with data from a single region, the L-DLPFC. Further research implementing approaches to account for multi-site/scanner studies, including statistical approaches and ComBat harmonization, that consider MRS data in different groups, brain regions, and acquisition protocols will provide important opportunities to replicate these results and explore the flexibility of these tools.

## 5. Conclusion

In a large clinical population, we found that different analysis techniques used to control for the site and scanner in MRS data could yield different results. Therefore, we recommend ensuring that there is consistency between single scanner data and different approaches to account for the scanner in multi-scanner studies. We have also demonstrated that ComBat harmonization can control for site (or vendor or scanner) effects in clinical MRS data.

## Data availability statement

A dataset with deidentified participant data and a data dictionary will be made available upon reasonable request from any qualified investigator, subject to a signed data access agreement.

## Ethics statement

This study was approved by the research ethics board at each participating site, and informed consent and assent was obtained from the parents/guardians and the youth participants respectively.

## Author contributions

PL: formal analysis, data curation, writing the first draft, and data visualization. PL, AH, and TB: conceptualization and methodology. AH: project administration and supervision. All authors contributed to data collection, reviewing, and editing the final submitted manuscript.
