# Supplementary material for: Comparison of different approaches to manage multi-site magnetic resonance spectroscopy clinical data analysis
Source: Front Psychol. 2023 Apr 20;14:1130188. doi: 10.3389/fpsyg.2023.1130188 (PMC10157208; doi:10.3389/fpsyg.2023.1130188)
Supplement: Supplementary file 1 [file Data_Sheet_1.docx]

Supplementary Material

Comparison of different approaches to manage multi-site MRS clinical data analysis

**Parker L La^*^, Tiffany K. Bell, William Craig, Quynh Doan, Miriam H. Beauchamp, Roger Zemek, Pediatric Emergency Research Canada (PERC), Keith Owen Yeates, Ashley D Harris**

*** Correspondence:** Corresponding Author: parker.la@ucalgary.ca

**_
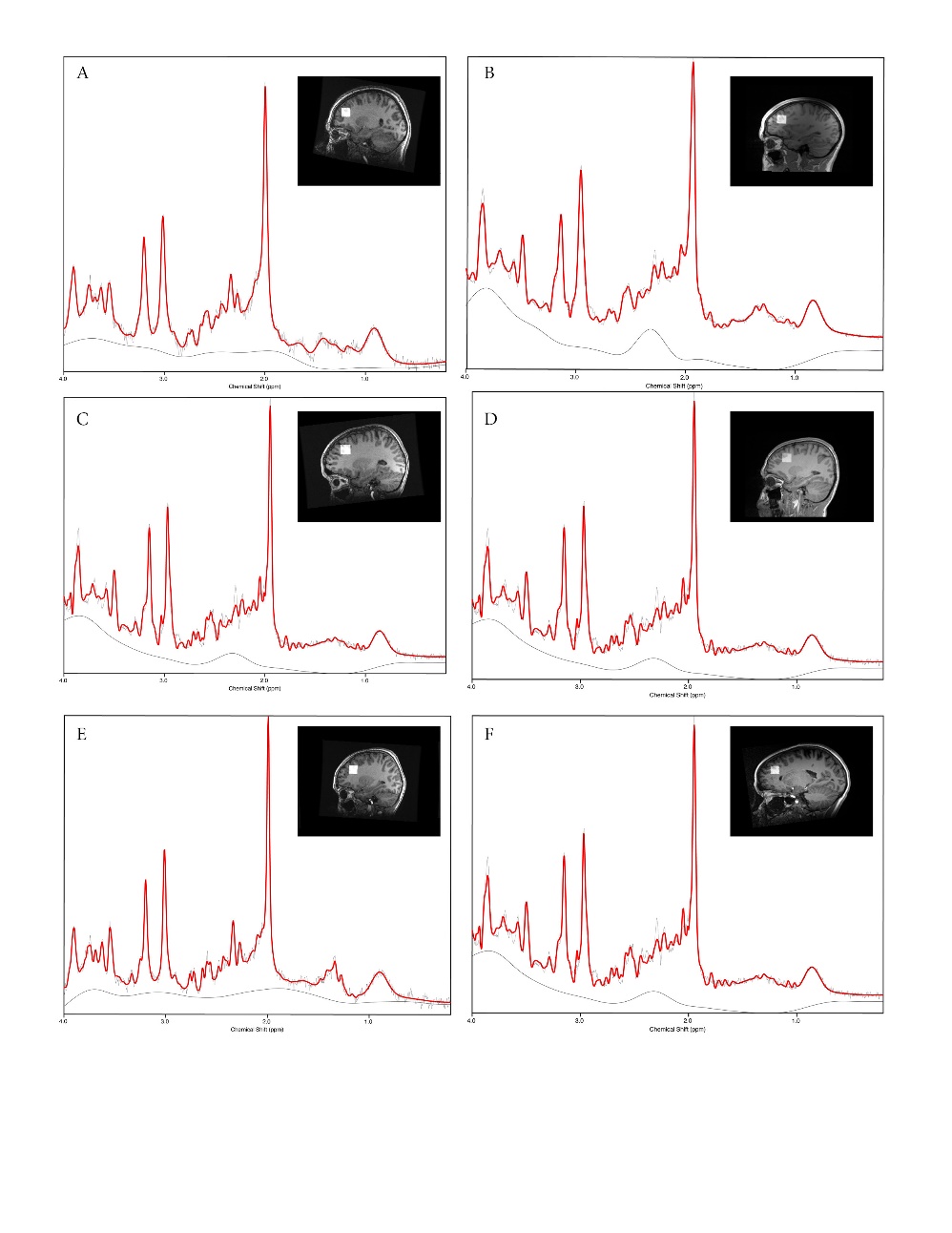
_**

**Supplementary Figure 1.** Example MRS spectrum of participant data at each site A) Calgary GE, B) Edmonton Siemens, C) Montreal GE, D) Montreal Siemens, E) Vancouver GE, and F) Ottawa Siemens. Data was analyzed with LCModel. The voxel placement was in the Left-Dorsal Lateral Pre-frontal Cortex. The voxel size was 2$\times$2$\times$2 cm^3^.­­


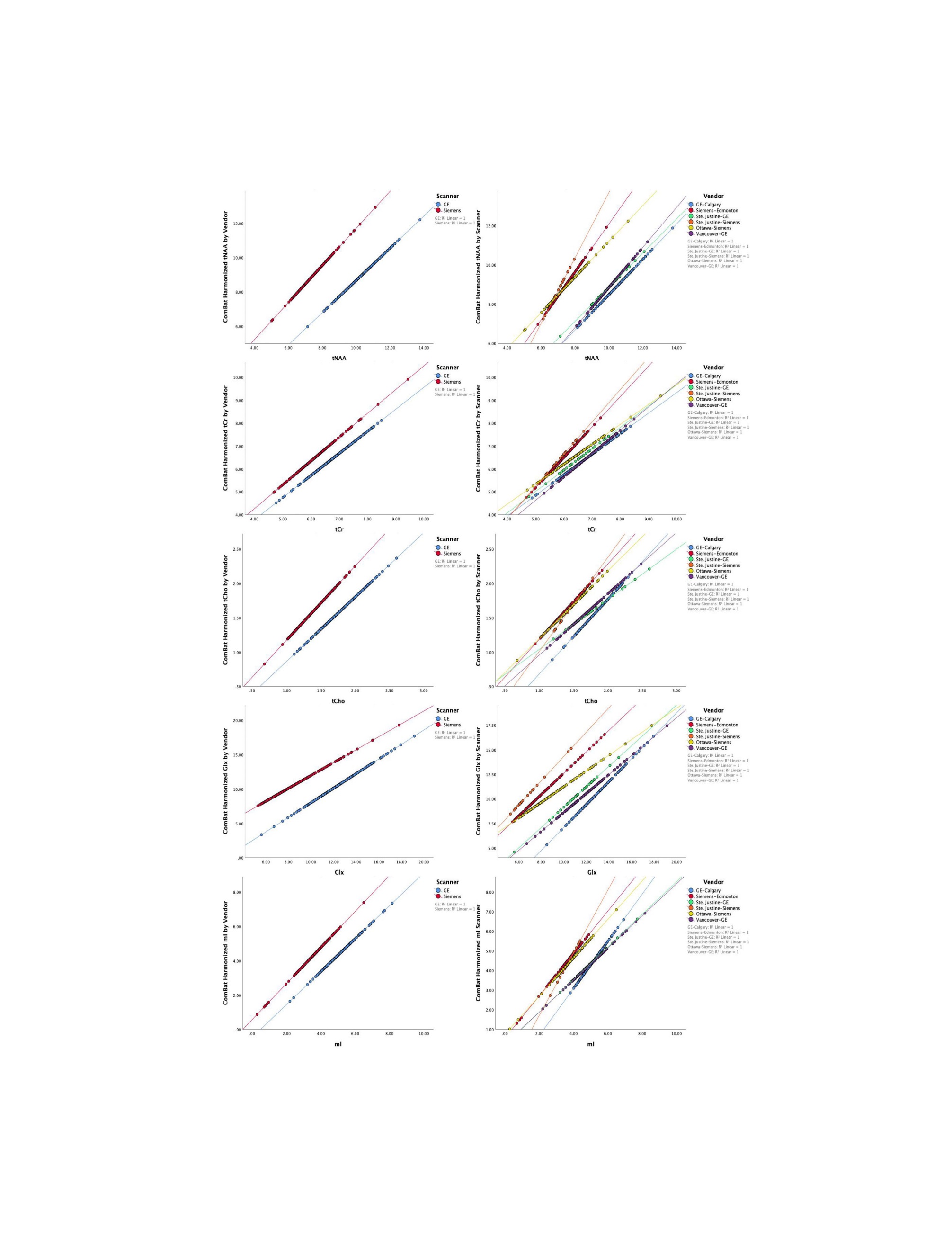


**Supplementary Figure 2.** Scatterplots describing the relationship between quantified metabolite concentrations (tNAA, tCr, tCho, Glx, and mI) and the resulting ComBat harmonization of the quantified metabolite concentrations by vendor or scanner.

**Supplementary Table 1.** Minimum reporting standards for in vivo magnetic resonance spectroscopy studies.

| Site (Name or Number) | Calgary | Edmonton | Montreal 1 | Montreal 2 | Ottawa | Vancouver |
| --- | --- | --- | --- | --- | --- | --- |
| 1. Hardware |  |  |  |  |  |  |
| a. Field strength [T] | 3 | 3 | 3 | 3 | 3 | 3 |
| b. Manufacturer | GE | Siemens | GE | Siemens | Siemens | GE |
| c. Model (software version if available) | GE MR750w | Siemens Prisma | GE MR750 | Siemens Prisma | Siemens Skyra | GE MR750 |
| d. RF coils: nuclei (transmit/ receive), number of channels, type, body part | ^1^H, 32-channel head coil | ^1^H, 64-channel head/neck coil | ^1^H, 32-channel head coil | ^1^H, 64-channel head coil | ^1^H, 64-channel head coil | ^1^H, 32-channel head coil |
| e. Additional hardware | No | No | No | No | No | No |
| 2. Acquisition |  |  |  |  |  |  |
| a. Pulse sequence | Vendor-supplied PRESS | Vendor-supplied PRESS | Vendor-supplied PRESS | Vendor-supplied PRESS | Vendor-supplied PRESS | Vendor-supplied PRESS |
| b. Volume of Interest (VOI) locations | Left Dorsallateral prefrontal cortex | Left Dorsallateral prefrontal cortex | Left Dorsallateral prefrontal cortex | Left Dorsallateral prefrontal cortex | Left Dorsallateral prefrontal cortex | Left Dorsallateral prefrontal cortex |
| c. Nominal VOI size [cm^3^] | 222  | 222  | 222  | 222  | 222  | 222  |
| d. Repetition Time (TR), Echo Time (TE) [ms] | 2000/30 | 2000/30 | 2000/30 | 2000/30 | 2000/30 | 2000/30 |
| e. Total number of Excitations or acquisitions per spectrum  In time series for kinetic studies   1. Number of Averaged spectra (NA) per time-point 2. Averaging method (e.g. block-wise or moving average) 3. Total number of spectra (acquired / in time-series) | 96 averages with an 8-step phase cycle | 96 averages with a 12-step phase cycle | 96 averages with an 8-step phase cycle | 96 averages with an 8-step phase cycle | 96 averages with an 8-step phase cycle | 96 averages with an 8-step phase cycle |
| f. Additional sequence parameters  (spectral width in Hz, number of spectral points, frequency offsets) | 5000 Hz, n=4096 | 2000 Hz, n=2048 | 5000 Hz, n=4096 | 2000 Hz, n=2048 | 2000 Hz, n=2048 | 5000 Hz, n=4096 |
| g. Water Suppression Method | CHESS (vendor provided) | WET (vendor provided) | CHESS (vendor provided) | WET (vendor provided) | WET (vendor provided) | CHESS (vendor provided) |
| h. Shimming Method, reference peak, and thresholds for “acceptance of shim” chosen | Automated B0 field mapping (vendor provided). No threshold was specified. | Automated B0 field mapping (vendor provided). No threshold was specified. | Automated B0 field mapping (vendor provided). No threshold was specified. | automated B0 field mapping (vendor provided). No threshold was specified. | Automated B0 field mapping (vendor provided). No threshold was specified. | Automated B0 field mapping (vendor provided). No threshold was specified. |
| i. Triggering or motion correction method  (respiratory, peripheral, cardiac triggering, incl. device used and delays) | N/A | N/A | N/A | N/A | N/A | N/A |
| 3. Data analysis methods and outputs |  |  |  |  |  |  |
| a. Analysis software | LCModel 6.3-1J | LCModel 6.3-1J | LCModel 6.3-1J | LCModel 6.3-1J | LCModel 6.3-1J | LCModel 6.3-1J |
| b. Processing steps deviating from quoted reference or product | Data pre-processed through FID-A | Data converted to .RAW format from FID-A prior to quantification in LCModel | Data pre-processed through FID-A | Data converted to .RAW format from FID-A prior to quantification in LCModel | Data converted to .RAW format from FID-A prior to quantification in LCModel | Data pre-processed through FID-A |
| c. Output measure  (e.g. absolute concentration, institutional units, ratio)Processing steps deviating from quoted reference or product | Absolute Concentration | Absolute Concentration | Absolute Concentration | Absolute Concentration | Absolute Concentration | Absolute Concentration |
| d. Quantification references and assumptions, fitting model assumptions | Custom basissets based on vendor-specific waveforms, generated from FID-A | Custom basissets based on vendor-specific waveforms, generated from FID-A | Custom basissets based on vendor-specific waveforms, generated from FID-A | Custom basissets based on vendor-specific waveforms, generated from FID-A | Custom basissets based on vendor-specific waveforms, generated from FID-A | Custom basissets based on vendor-specific waveforms, generated from FID-A |
| 4. Data Quality |  |  |  |  |  |  |
| a. Reported variables  (SNR, Linewidth (with reference peaks)) | SNR, Linewidth (tNAA) | SNR, Linewidth (tNAA) | SNR, Linewidth (tNAA) | SNR, Linewidth (tNAA) | SNR, Linewidth (tNAA) | SNR, Linewidth (tNAA) |
| b. Data exclusion criteria | Excessive motion, low SNR, high linewidth | Excessive motion, low SNR, high linewidth | Excessive motion, low SNR, high linewidth | Excessive motion, low SNR, high linewidth | Excessive motion, low SNR, high linewidth | Excessive motion, low SNR, high linewidth |
| c. Quality measures of postprocessing Model fitting (e.g. CRLB, goodness of fit, SD of residual) | CRLB, SNR, Linewidth | CRLB, SNR, Linewidth | CRLB, SNR, Linewidth | CRLB, SNR, Linewidth | CRLB, SNR, Linewidth | CRLB, SNR, Linewidth |
| d. Sample Spectrum | Yes, supplementary figure 1 | Yes, supplementary figure 1 | Yes, supplementary figure 1 | Yes, supplementary figure 1 | Yes, supplementary figure 1 | Yes, supplementary figure 1 |

**Supplementary Table 2.** MRS quality assurance measures (Means and standard deviation (SD) of Full-Width Half-Maximum (FWHM measured on the water peak) and Signal-to-Noise Ratio (SNR measured on the tNAA peak) of all the scanners.

|  | GE-Calgary | Siemens-Edmonton | GE-Montreal | Siemens-Montreal | Siemens-Ottawa | GE-Vancouver |
| --- | --- | --- | --- | --- | --- | --- |
|  | Mean ± SD | Mean ± SD | Mean ± SD | Mean ± SD | Mean ± SD | Mean ± SD |
| **FWHM** | 11.09 ± 2.24 | 8.05 ± 4.45 | 7.82 ± 1.33 | 8.74 ± 4.78 | 8.59 ± 4.96 | 10.61 ± 4.04 |
| **SNR** | 77.67 ± 10.89 | 203.16 ± 33.01 | 98.67 ± 22.35 | 207.01 ± 35.66 | 161.09 ± 34.5 | 84.77 ± 21.61 |

**Supplementary Table 3.** Metabolite concentrations of each metabolite divided by scanner and group. Mean concentrations and standard deviations (SD) are provided.

|  | **GE-Calgary** | | **Siemens-Edmonton** | | **GE-Montreal** | | **Siemens- Montreal** | | **Siemens- Ottawa** | | **GE-Vancouver** | |
| --- | --- | --- | --- | --- | --- | --- | --- | --- | --- | --- | --- | --- |
|  | Concussion | OI | Concussion | OI | Concussion | OI | Concussion | OI | Concussion | OI | Concussion | OI |
|  | Mean ± SD | Mean ± SD | Mean ± SD | Mean ± SD | Mean ± SD | Mean ± SD | Mean ± SD | Mean ± SD | Mean ± SD | Mean ± SD | Mean ± SD | Mean ± SD |
| **tNAA** | 10.48 ± .93 | 10.39 ± .88 | 7.37 ± .72 | 7.41 ± .59 | 10.21 ± .79 | 9.17 ± 1.24 | 7.02 ± .51 | 7.32 ± .43 | 7.40 ± .88 | 7.66 ± .97 | 9.97 ± .70 | 10.11 ± .91 |
| **tCr** | 6.80 ± .70 | 6.72 ± .64 | 5.97 ± .46 | 5.90 ± .47 | 6.62 ± .65 | 6.08 ± .48 | 5.79 ± .44 | 5.97 ± .42 | 6.21 ± .62 | 6.47 ± .93 | 6.77 ± .56 | 6.78 ± .64 |
| **tCho** | 1.82 ± .20 | 1.84 ± .22 | 1.35 ± .21 | 1.37 ± .20 | 1.69 ± .26 | 1.95 ± .48 | 1.42 ± .18 | 1.41 ± .15 | 1.38 ± .23 | 1.40 ± .22 | 1.67 ± .25 | 1.72 ± .26 |
| **Glx** | 12.88 ± 1.6 | 12.9 ± 1.56 | 8.00 ± 1.69 | 8.08 ± 1.73 | 11.6 ± 1.47 | 9.59 ± 2.34 | 6.98 ± 1.67 | 6.72 ± 1.11 | 9.08 ± 2.36 | 8.81 ± 2.17 | 11.9 ± 1.83 | 11.92 ± 2.01 |
| **mI** | 5.03 ± .57 | 5.16 ± .67 | 3.56 ± .61 | 3.45 ± .92 | 5.20 ± .83 | 4.18 ± .65 | 3.67 ± .50 | 3.68 ± .38 | 3.71 ± .66 | 3.79 ± 1.02 | 5.04 ± .87 | 5.09 ± .92 |
