# Supplementary figures and images for: Comparison of different approaches to manage multi-site magnetic resonance spectroscopy clinical data analysis
Source: Front Psychol. 2023 Apr 20;14:1130188. doi: 10.3389/fpsyg.2023.1130188 (PMC10157208; doi:10.3389/fpsyg.2023.1130188)

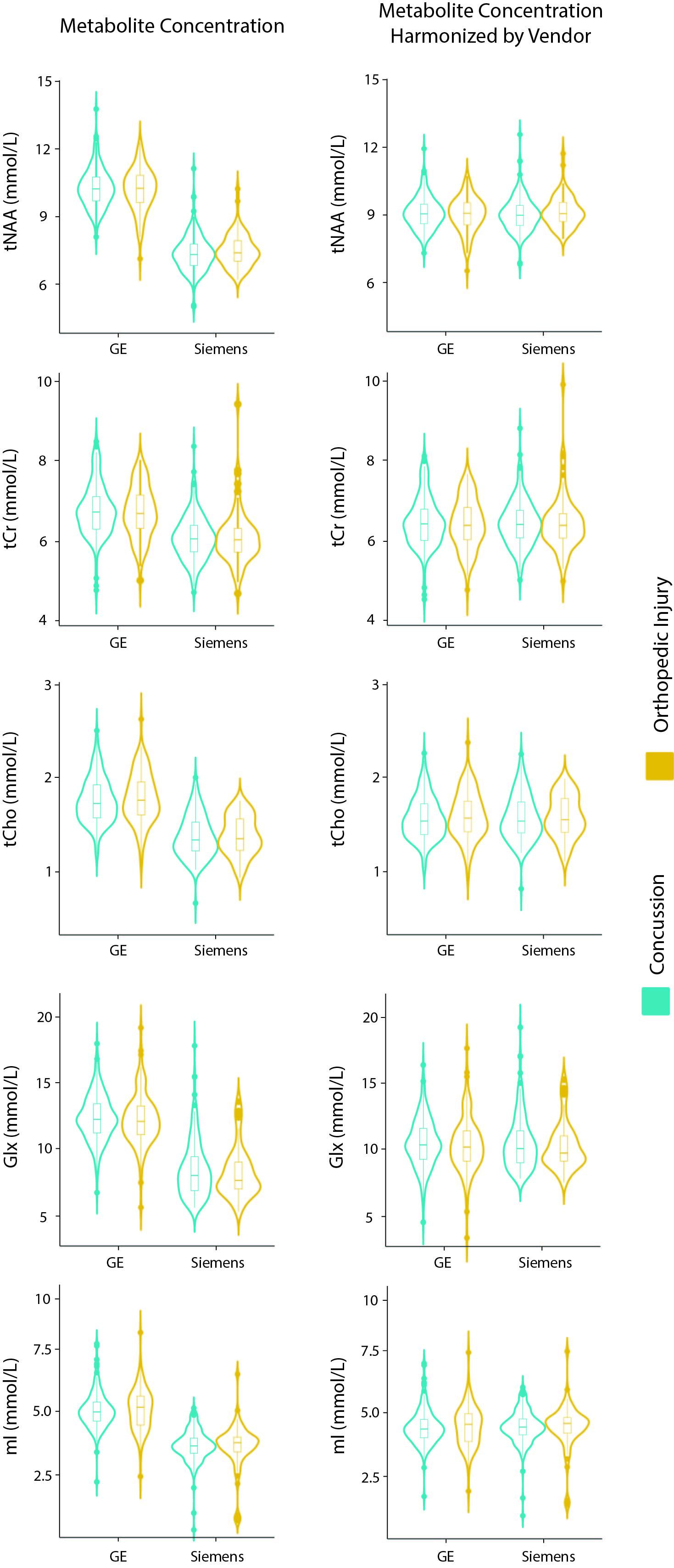

Supplement: Supplementary file 2 [file Figure_1.JPEG]

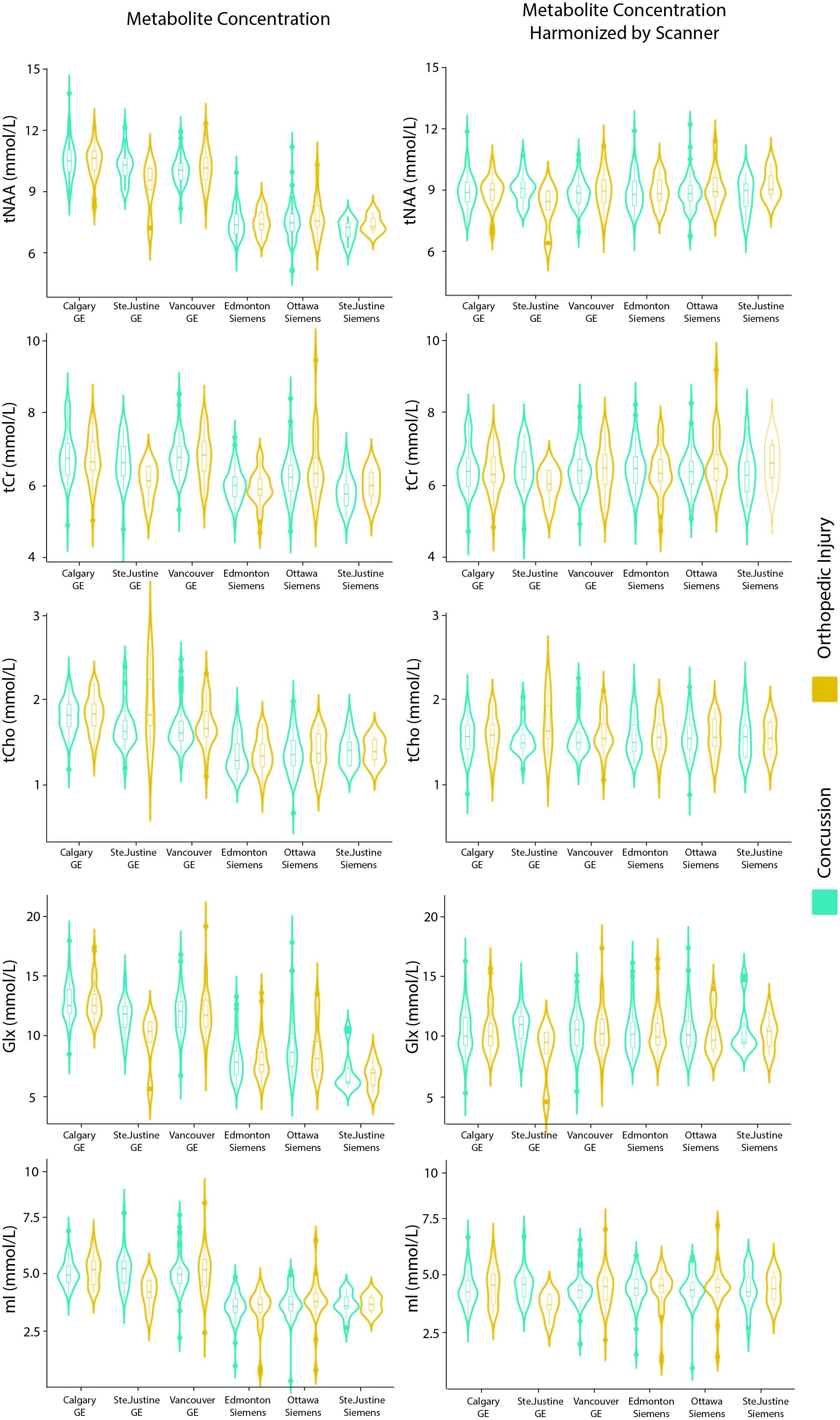

Supplement: Supplementary file 3 [file Figure_2.JPEG]
